# Regulation of Neutral Amino Acid Transport By the SARS-CoV-2 Receptor ACE2

**DOI:** 10.1093/function/zqab048

**Published:** 2021-09-04

**Authors:** Donald D F Loo, Ernest M Wright

**Affiliations:** Department of Physiology, The Geffen School of Medicine at UCLA, Los Angeles, CA 90095-1751, USA; Department of Physiology, The Geffen School of Medicine at UCLA, Los Angeles, CA 90095-1751, USA

## A Perspective on “B^0^AT1 Amino Acid Transporter Complexed With SARS-CoV-2 Receptor ACE2 Forms Heterodimer Functional Unit: In Situ Conformation Using Radiation Inactivation Analysis”

Ongoing research continually uncovers surprises in the roles for membrane-bound proteins including transporters. Classical era studies of amino acid transporters focused on classifications by substrate selectivity, voltage, and ion dependency. Landmark studies by Stevens and colleagues, using intestinal brush-border and basolateral membrane vesicles, identified sodium-dependent transporters including the major neutral amino acid (NBB, now known as B^0^AT1) and proline selective (Imino) transporters.^1^ Following the expression cloning of intestinal sodium–glucose cotransporter SGLT1 in 1987,^2^ sodium-dependent transporters including B^0^AT1 and Imino (SIT1) were cloned and expressed in *Xenopus laevis* oocytes and cultured cells.^3^^,^^4^ B^0^AT1 (SLC6A19) and SIT1 (SLC6A20) are members of the large SLC6 family that includes sodium-dependent neurotransmitters, with a separate clade of nutrient amino acid transporters mainly expressed in the intestine, kidney, and/or brain.^4–6^

It was discovered that B^o^AT1 was regulated by tissue-specific apical membrane expression of two members of the Renin Angiotensin System (RAS): collectrin in the kidney; and angiotensin-converting enzyme 2 (ACE2) in the intestine.^6^ Collectrin is a 50% homologue of ACE2 sharing identity with the non-catalytic, transmembrane helix but lacks the ACE2 carboxypeptidase ectodomain. In the oocyte expression system, collectrin and ACE2 increase trafficking of B^0^AT1 to the plasma membrane and modulate transporter activity. The effect of intestinal ACE2 on transport is independent of its function as a carboxypeptidase. Much clinical interest in the regulation of B^0^AT1 in the intestine and kidney stems from mutations that cause Hartnup disease.^3^^,^^6^^,^^7^

**Figure 1.**
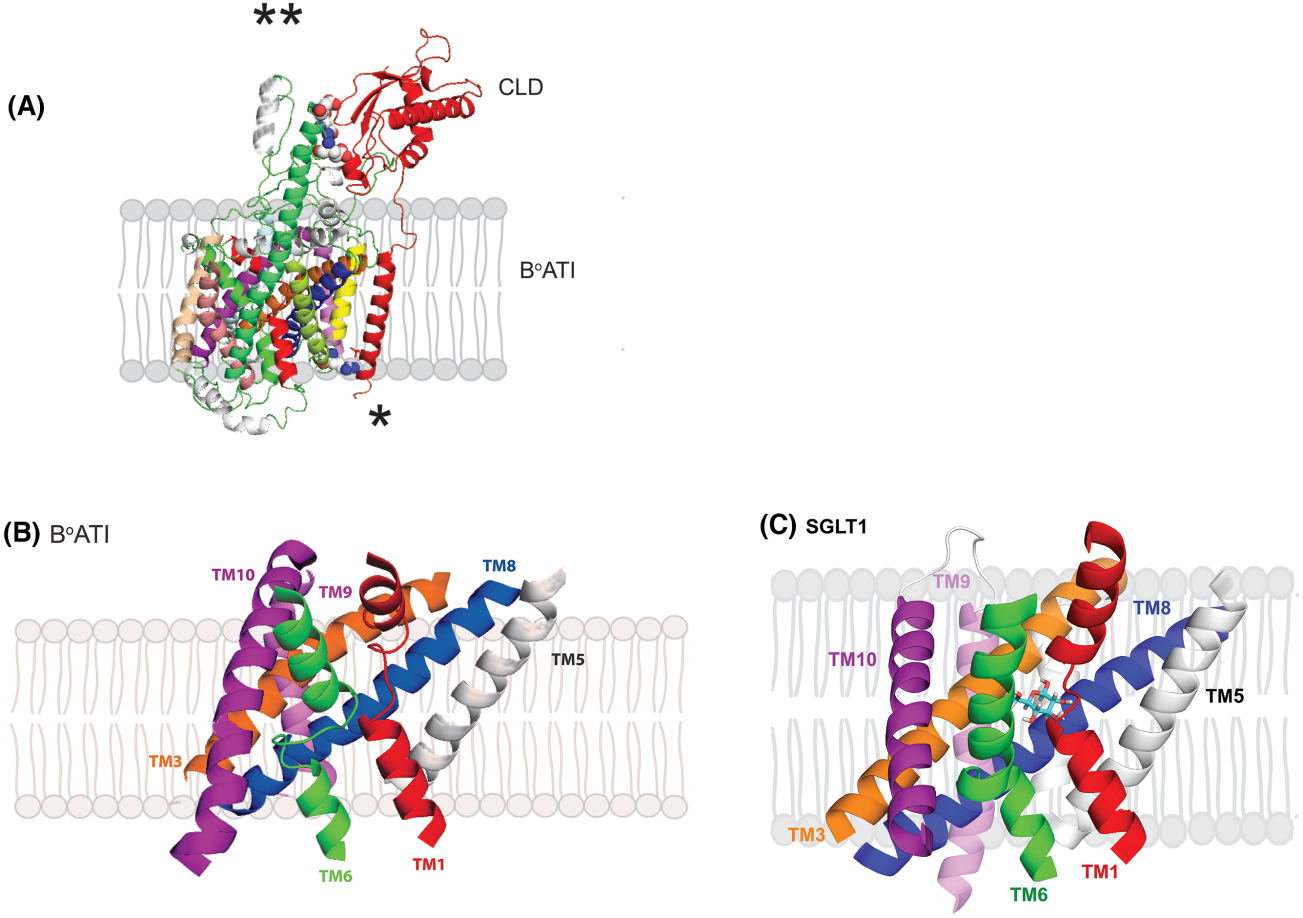
Heterodimer formed by B^0^AT1 and ACE2 in intestinal brush border membranes. (A) The interaction between B^0^AT1 and the collectrin-like (CLD) membrane-anchoring domain of ACE2 (highlighted in red; not shown is the ACE2 region that binds SARS-CoV-2). Note the long extra-cellular extension of TM7 (green helix) interacts with the CLD. ** the site of H-bonding between the neck region of ACE2 and TM7 of B^0^AT1 (K676 of ACE2 and D349 of B^0^AT1, R678 of ACE2 and N346 of B^0^AT1, and K625 of ACE2 and E352 of B^0^AT1). * The site of the H-bonding between the ACE2 transmembrane membrane anchor and B^0^AT1 (L760 of ACE2 and R214 of B^0^AT1). (B) B^0^AT1 membrane structural fold. (C) SGLT1 membrane structural fold. In (B) and (C), TM helices 2, 4, 5, and 7 are removed for clarity. Both B^0^AT1 and SGLT1 have a core structure of 10 TM helices arranged in a 5 TM inverted repeat with unwound regions of TM1 and TM6 forming the substrate binding site in the center of the membrane. B^0^AT1 and SGLT1 are shown in the outward open conformation. The structures of (A) and (B) were obtained from PDB: 6M18, and (C) taken from reference 2.

Considerable biochemical evidence has been gathered showing that B^0^AT1 and ACE2 are co-expressed in intestinal brush-border membranes, but the nature of the interactions was not known. This changed dramatically in 2020 with the publication of the cryo-EM high resolution structure of B^0^AT1 complexed with ACE2 expressed and purified from HEK293F cells.^8^ The complex is assembled as a 2[ACE2: B^0^AT1] dimer-of-heterodimers (See, Figure 1 in reference 8; and Figure 4 in reference 9). Apart from ACE2’s function as the SARS-CoV-2 receptor, and the importance of co-expressing B^0^AT1 with ACE2 in the screening development of a COVID-19 mRNA vaccine, the 3D atomic coordinates raised provocative questions about the functional interaction of ACE2 and B^0^AT1. Such questions include how does ACE2 govern B^0^AT1 brush border expression and activity.

Stevens and colleagues addressed this question directly in intestinal brush borders by deploying the well-established method of radiation inactivation, as reported in the literature to determine the functional unit of various enzymes, receptors, channels, and transporters.^9^ They assayed sodium-dependent alanine and serine transport in rabbit brush border membrane vesicles before and after exposing the membranes to varying doses of high energy electrons. Then using electron flux targeting theory developed by Ellis Kempner, they estimated the molecular size of the functional unit responsible for neutral amino acid transport activity and used molecular modelling to locate the residues involved in interface contact between subunits. The functional molecular size of the B^0^AT1: ACE2 complex was 184 kDa—the expected sum of B^0^AT1 and ACE2 subunits in a heterodimer, which was half the 345 kDa size of the dimer-of-heterodimers complex (Figure 1A). Furthermore, their molecular modelling predicted the location, identity, and distance of pairs of resides involved in heterodimer interface contacts: three extracellular pairs between B^0^AT1 TM7 and the neck of the non-catalytic membrane-anchoring domain of ACE2; and one pair between B^0^AT1 TM4 and the transmembrane domain of ACE2 (Figure 1A).

Unlike the intestine, the kidney expresses the ACE2 homologous variant, collectrin, which lacks the carboxypeptidase ectodomain. This immediately raises questions about how ACE2 and collectrin differentially regulate B^0^AT1 expression and activity in the kidney, intestine, and in vitro expression systems. Is B^0^AT1 expression in the brush border surface determined by ACE2 or collectrin interactions in the ER, Golgi, and/or trafficking vesicles?

What can we learn about the function of B^0^AT1 from the three high-resolution cryo-EM structures (PDB files 6M1D, 6M17, and 6M18)? First, its transmembrane structural fold matches that of transporters in the APC superfamily (2A.21) in the Transporter Classification Database (http://www.tcdb.org) that includes SLC5 and SLC6 sodium-cotransporters. Here, a core of 10 TM helices is arranged in an inverted repeat, TM1-5, TM6-10, with two helices, TM1 and TM6, unwound in the middle of the membrane (see Figure 1B and C). Substrates bind to side chains of residues on these unwound helices, and transport involves an alternate-access system whereby (i) external substrates enter the binding site, (ii) an external gate closes to occlude the substrate before (iii) an internal gate opens to release substrate to the cytoplasm.^2^^,^^10^ We conclude that the current structure of B^0^AT1 is in an outward open conformation.

A unique aspect of the B^0^AT1 structure is the long external extension of TM7 (green helix in Figure 1A), where the tip binds to the neck of the CLD of ACE2; this motif is shared with other members of the SLC6 clade: B^o^AT2 (SLC6A15), NTT4 (SLC5A16), and B^o^AT3 (SLC6A17). B^0^AT1 (SLC5A19) and SIT1 (Imino, SLC5A20). The motif is most likely involved in regulation of their activity by ACE2 in the intestine and collectrin in the kidney.

Solving the structure of B^0^AT1 complexing with the COVID-19 receptor ACE2 not only provided important advances for the development of mRNA vaccines, but it revealed the structural interactions between ACE2 and B^0^AT1 underlying its regulation of amino acid transport in the intestine and kidney. However, structures require functional investigation. Stevens and colleagues used functional studies to firmly establish that B^0^AT1: ACE2 in intestinal brush borders is a heterodimer and highlighted the residue contacts between the collectrin-like-domain of intestinal ACE2 that interacts with B^0^AT1. This opens the way to functional studies to determine how these contacts determine B^0^AT1 trafficking to the brush border and regulate transporter activity. These obviously include mutation of key residues in B^0^AT1 TM7 and the collectrin domain of ACE2, their expression in oocytes and cultured cells, and biophysical studies such as those pioneered for SGLT1.^2^

## Funding

None declared.
